# Evaluating an e-learning course's impact and challenges on genomic literacy among medical professionals

**DOI:** 10.5116/ijme.6736.4367

**Published:** 2024-11-22

**Authors:** Miwa Arita, Takami Maeno, Emiko Noguchi, Motoko Sasaki, Hidehiko Miyake

**Affiliations:** 1 Department of Clinical Genetics, University of Tsukuba Hospital, Japan; 2 Department of General Medicine and Primary Care, University of Tsukuba Hospital, Japan; 3 Department of Genetic Counseling, Division of Life Sciences, Graduate School of Humanities and Sciences, Ochanomizu, University, Tokyo, Japan

**Keywords:** Genomic education programme, e-learning, medical professionals, genetic medicine, genomic literacy

## Abstract

**Objectives:**

The aim of this study was to confirm and evaluate the learning effect of a physician-facing e-learning course on genetic medicine for improving genomic literacy.

**Methods:**

We employed qualitative and quantitative methodology to survey 103 physicians who took the course at a national university in Japan. Evaluations were conducted at the levels of participant feedback, learning, and behaviour. Participants completed a questionnaire and test (full score = 100) before and after the course. Pre- and post-test scores were compared using paired-samples t-tests and Mann-Whitney U test was used to compare the difference their clinical experience. The effect size was estimated using Cohen’s d.

**Results:**

Responses were obtained from 96 physicians. Approximately 80% (n = 75-93) of participants responded positively to the course, a result supported by the qualitative data. The mean scores for the pre- and post-test showed an increase from 71.25 to 74.58 (p=0.008). In particular, mean test scores increased significantly from 68.94 to 75.53 (p<0.001) in physicians with no clinical experience in genetic medicine, while no significance was observed scores for physicians with clinical experience in genetics from 73.47 to 73.67 (p=0.903). Behavioural assessment was carried out for 28 participants; however, no statistically significant differences were identified.

**Conclusions:**

Our findings indicate that our e-learning course was useful for physicians with no experience of genetic medicine. For those with experience, it may be necessary to provide more practice-based education and educational methodologies. Behavioural assessment needs to be examined further.

## Introduction

Recent advancements in medical genetics and genomic medicine have resulted in a significant expansion of their application in day-to-day clinical practice and a subsequent increasing demand for genomic literacy among healthcare professionals. To improve genomic literacy, defined as ‘the capacity to obtain, process, understand, and use genomic information for health-related decision-making’,^1^^,^^2^ many countries hold workshops for healthcare workers, have online information repositories for the general public and rare disease patients and families, and develop guidelines, standards, and national programmes for implementation of genomic education into formal education.^3^ The advancements in medical genetics and genomic medicine have necessitated all medical professionals to be proficient in genetic treatment and understand the pertinent ethical issues; indeed, education in medical genetics is becoming increasingly important in undergraduate education.^4^

While genetic information requires ethical consideration and appropriate information management owing to its private and sensitive nature, as medical information, it also needs to be shared by medical professionals.^5^ Therefore, it is essential that medical professionals have the opportunity to learn the ethical issues and to practice appropriate genetic medicine. However, there have traditionally been relatively few opportunities to study medical genetics as part of a typical medical education curriculum. To address both advances in genetics and genomics as well as trends in medical education, the Association of Professors in Human and Medical Genetics (APHMG) updated its medical school core curriculum in genetics in 2013.^6^ Various methods of genetic education have been examined, such as active learning materials and needs-based education, and reports on their effectiveness have been published.^7^ Simulation-based learning environments have been shown to improve the quality of medical education by allowing students to interact with patients and patient data in a virtual environment.^8^ The means for simulation-based exercises are diverse, from programmable simulator mannequins or computer-based virtual reality to nontechnical applications using methods such as a situation-based roleplaying, standardised patients, or case-based, problem-based learning.^8^ The simulation-based learning environment has been reported to increase students’ learning, intrinsic motivation, and self-efficacy, and to increase the perception perceived relevance of medical educational activities;^8^  however, working medical professionals often lack time to attend in-person classes. For busy medical professionals, e-learning is a very convenient mode of study as it allows them to study where and when they like. While there have been numerous reports on the learning effects of e-learning in medical education, few have evaluated medical genetics e-learning courses for health care professionals.

In Japan, the Model Core Curriculum for Medical Education, revised in 2016, added ‘genetic and genomic medicine’ to ‘physiological changes, pathogenesis, diagnosis, and treatment throughout the body’.^9^ Currently, medical educators are endeavouring to reform curricula from the viewpoint of competency-based medical education (CBME).^10^ The quality of learner assessment is very important to control quality regarding whether such competencies are achieved.^11^ In Japan, it is necessary to promote seamless training of physicians in undergraduate education and post-graduate clinical training;^12^ therefore, undergraduate and post-graduate education in genetic medicine is currently under consideration.^1^

Previous research on genetic education seminars for healthcare professionals in Japan reported that self-assessments of satisfaction and understanding improved among those who attended more than one session.^13^ In addition, methods for appropriately assessing outcomes have been explored in genetic medicine, including self-assessment systems and new methods for performance assessment.^4^ However, course evaluation is largely based on self-reported evaluation and less so on objective or behavioural evaluation. In addition, there are no reports on the effectiveness or evaluation of genetic education for physicians or other health professionals as post-graduate or professional education.

The University of Tsukuba Hospital conducted an e-learning course on genetic medicine with the aim of improving the genetic literacy of medical professionals. The learning objective of this course was to develop physician competency for understanding genetic medicine and handling of genetic information at our hospital and to acquire basic knowledge to perform genetic testing. The aim of this study is to evaluate the learning impact of our e-learning course on enhancing genomic literacy among physicians and to explore its efficacy in teaching appropriate genetic medicine practices.

## Methods

### Outline of the e-learning

University of Tsukuba Hospital began conducting its e-learning course on genetic medicine for medical professionals from 2019. The course comprises eight comprehensive sections: i) ‘Introduction to the Genetic Medicine Department,’ offering an overview of the practices within genetic medicine; ii) ‘Basic knowledge of genetic information,’ which covers the fundamentals of genes, genomes, and personal data; iii) ‘Basic knowledge to perform genetic testing,’ details the procedures and unique characteristics of genetic testing; iv) ‘Genetic testing and genetic counselling in practice,’ which explores clinical scenarios involving genetic counselling; v) ‘Genetic information and electronic medical record description,’ outlines the proper protocols for documenting medical information; vi) ‘How genetic information can be accessed for genetic testing,’ describes the specific methods used to access genetic data; vii) ‘Genetic testing,’ enumerates the various genetic tests available; and viii) ‘Considerations and guidelines for genetic medicine,’ which presents critical considerations and standards for practice in genetic medicine. The course lasted approximately 90 minutes.

### Study design and Participants

**Figure 1:**
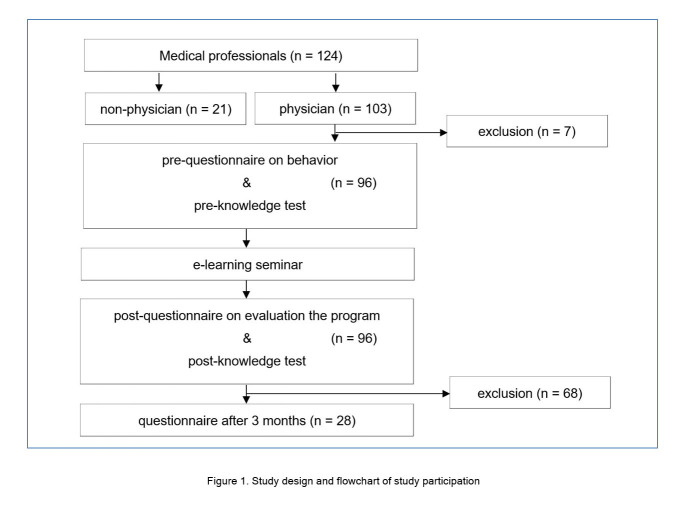
Study design and flowchart of study participation

This study was reviewed and exempted by the Institutional Review Board, Office of Human Research Ethics, University of Tsukuba Hospital. Study participants were medical professionals at the University of Tsukuba Hospital who were taking the e-learning course on genetic medicine. Based on Kirkpatrick’s four-stage evaluation concept,^14^^,^^15^ we conducted an evaluation of the e-learning course using participant feedback and assessment of learning and behaviour outcomes. Written informed consent was obtained from the participants. Before the e-learning course, participants completed a behavioural questionnaire and answered a test on their knowledge of genetic medicine (Appendix 1),(Appendix 2). After the course, the participants took the same test again and evaluated the course. Three months later, the participants completed a second behavioural questionnaire. Figure 1 provides information on the study design and flow of study participation.

Study participants completed the e-learning course and then completed a behavioural questionnaire three months later. They took a genetic medicine knowledge test before and after the e-learning course and evaluated the program upon completion. Self-assessment of the participants’ genetic practice behaviour before and three months after the e-learning was also carried out.

The study population comprised 124 medical professionals, of whom 103 were physicians, who took the e-learning course between January 2020 and September 2022. A total of 96 (93.2%) of these physicians were included in the analysis. Behavioural assessment was analysed using data gathered from 28 participants who responded to questionnaires both before and three months after the course. Exclusion criteria were those who did not respond to the pre- and post-course questionnaires and tests. Only physicians were included in this study.

### Data collection

In collecting data, we obtained cooperation from the Education Cloud Promotion Office, which was established to promote e-learning and improve the quality of education using Information and Communication Technology at the University of Tsukuba. A learning management system was used to record lectures, produce and distribute content, and electronically record e-learning attendance and response status (Appendix 3).

### Quantitative data

Participants evaluated the e-learning course across the following criteria: learning location, implementation time, length of the lecture, ease of reading text and slides, content of the lecture, clinical usefulness, and operation of the e-learning software. Responses were given using a 4-point Likert scale (1 = Poor, 2 = Fair, 3 = Good, and 4 = Very good). The participants then evaluated their understanding of the learning content for each of the eight chapters using a 4-point scale (1 = Did not understand at all, 2 = Understand somewhat, 3 = Understood well, 4 = Understood very well). They completed pre- and post-test to measure any changes in genetic medicine knowledge due to the e-learning course. The degree of mastery was scored in terms of the number of correct answers to 10 questions that pertained to ethical considerations of genetic. The questions test were prepared by referring to the Act on the Protection of Personal Information ^16^and the Medical Fee Score Table,^17^ and were discussed and prepared by the co-researchers, several genetic specialists outside the hospital, general medical staff, and general medical staff in the hospital. Behavioural evaluations with regards genetic medicine-related actions/behaviour were conducted using a self-assessment before and three months after completion of the e-learning course. Respondents answered either ‘Yes’ or ‘No’ for each item of the behavioural questionnaire. The questionnaire was prepared by several medical professionals involved in genetic medicine, based on the guidelines on genetic testing and diagnosis in medicine^5^and the hospital’s manual on the prevention of medical accidents.

### Qualitative data

A self-administered, open-ended questionnaire was conducted after the e-learning course in which participants were asked to write comments and opinions on the management of genetic information, workshops, and the e-learning course.

### Data analysis

#### Quantitative data

The self-assessment scores for the genetic medicine knowledge test are expressed as means with corresponding standard deviation (SD). Pre- and post-test scores were compared using paired-samples t-tests. Mann-Whitney U test was used to compare the difference between physicians with clinical experience in genetic medicine and those without. The mean test scores of physicians with and those without such experience were compared using independent t-tests.

Pearson correlation was used to determine the association between two numerical variables. Univariate and multivariate analyses were conducted using the regression model to evaluate potential confounders. Multivariate regression analysis was used to evaluate the contribution of the measured parameters to increase in pre- to post-test scores as the dependent variable.

All statistical tests were two-sided, and p-values of ≤0.05 were deemed to be of statistical significance. The effect size was calculated to demonstrate the strength of differences between pair measures and estimated using Cohen’s d. All statistical analyses were conducted using SPSS 28.0 (IBM Corporation, Armonk, New York, USA).

#### Qualitative data

Qualitative analysis of the written responses was performed to generate emergent themes.

## Results

The characteristics of the participants (physicians) are listed in Table 1. Of these participants, 51% (n = 49) had clinical experience in genetic medicine and paediatricians accounted for the highest proportion (31.3%; n = 30). The time required to implement the e-learning course varied from ≤15 to 90 minutes, with the highest percentage of respondents taking 30-60 minutes (55.8%; n = 53).

**Table 1:** Participants characteristics (n=96)

Variable		n (%)
Gender		
	Male	60 (62.5)
	Female	36 (37.5)
Age		
	20s	9 (9.4)
	30s	43 (44.8)
	40s	31 (32.3)
	50s	13 (13.5)
	60s	0 (0)
Years of clinical experience		
	<5 years	12 (12.5)
	5-10 years	23 (24.0)
	10-15 years	25 (26.0)
	15-20 years	14 (14.6)
	20-25 years	9 (9.4)
	25-30 years	6 (6.3)
	>30 years	4 (4.2)
	No record	3 (3.1)
Department		
	Paediatrics	30 (31.3)
	Obstetrics and Gynaecology	9 (9.4)
	Breast-Thyroid-Endocrine Surgery	8 (8.3)
	Neurology	7 (7.3)
	Endocrinology and Metabolism	5 (5.2)
	Gastroenterology	4 (4.2)
	Cardiology	4 (4.2)
	Radiology	3 (3.1)
	Oncology	2 (2.1)
	Haematology	2 (2.1)
	Urology	2 (2.1)
	Dermatology	2 (2.1)
	Other	6 (6.3)
	No record/training doctor	10 (10.4)
	Clinical Geneticist	6 (6.3)
Clinical experience in genetic medicine		
	With clinical experience in genetic medicine	49 (51.0)
	Without clinical experience in genetic medicine	47 (49.0)
Years of experience in genetic medicine		
	No experience	47 (49.0)
	<5 years	24 (25.0)
	5-10 years	9 (9.4)
	10-15 years	7 (7.3)
	15-20 years	6 (6.3)
	>21 years	3 (3.1)

### Participant feedback

Approximately 80% (n = 75-93) of respondents expressed positive opinions (Good/Very good) regarding the content of the e-learning course and its usefulness for medical practice (see Table 2).

### Learning assessment

As presented in Table 3, Mann-Whitney U testing indicated no significant differences in the self-reported scores pertaining to understanding of genetic medicine between physicians with and without experience in genetic medicine. For example, physicians with clinical experience (Mdn=3.24) underperformed those who had not it (Mdn=3.30) on the self-reported scores of Basic knowledge of genetic information (U=1104.5, p=0.669)

**Table 2:** Frequency distribution and percentiles of physicians’ evaluation of the e-leaning programme (n=96)

e-learning	Poor (%)	Fair (%)	Good (%)	Very good (%)
Learning location	2 (2.1)	6 (6.3)	57 (59.4)	31 (32.3)
Implementation time	2 (2.1)	3 (3.2)	59 (62.1)	31 (32.6)
Length of the lecture	1 (1.0)	4 (4.2)	61 (63.5)	30 (31.3)
Ease of reading text and slides	2 (2.1)	3 (3.2)	52 (54.7)	38 (40)
Content of the lecture	2 (2.1)	1 (1.1)	51 (54.3)	40 (42.6)
Clinical usefulness	2 (2.1)	1 (1.0)	55 (57.3)	38 (39.6)
Operation of e-learning	4 (4.3)	15 (16.0)	51 (54.3)	24 (25.5)

**Figure 2:**
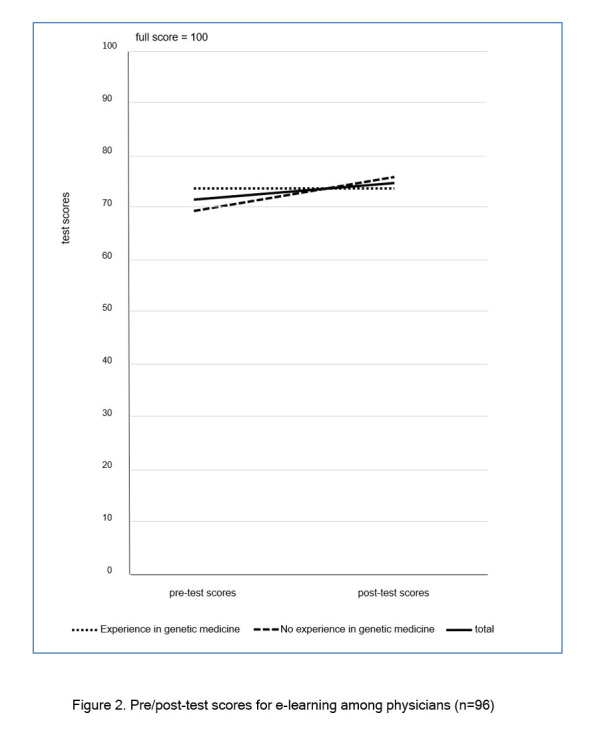
Pre/post-test scores for e-learning among physicians (n=96)

The mean scores for the pre- and post-tests regarding genetic medicine knowledge were 71.25 and 74.58, respectively; a significant increase (t _(95)_ =2.70, p=0.008), with a small-medium effect size (d=0.275). For physicians with no clinical experience in genetic medicine, the pre- and post-test scores increased significantly from 68.94 to 75.53 (t_(46)_=3.82, p<0.001), respectively, with a medium effect size (d=0.557). For those with clinical experience, scores did not differ significantly from 73.47 to 73.67 (t_(48)_ =0.12, p=0.903), respectively, with a small effect size (d=0.018). In addition, Mann-Whitney U test revealed that physicians with clinical experience in genetic medicine (Mdn=73.47) significantly outperformed those without (Mdn=68.94) on pre-test scores (U=1424.5, p=0.037). Meanwhile, physicians with clinical experience in genetic medicine (Mdn=73.67) did not outperform those without (Mdn= 75.53) on post-test scores (U=1056.5, p=0.466) (see Figure 2).

With or without clinical experience in genetic medicine, years of clinical experience, gender, and pre-test scores were used as independent variables in the multivariate regression analysis. According to the results of the multivariate regression with the increase in pre- to post-test scores as the objective variable, with or without clinical experience in genetic medicine (β=−0.208, t=2.21, p=0.03) and pre-course test scores (β=−0.459, t=4.91, p<0.001) were significantly and negatively associated, while years of clinical experience (β=0.188, t=2.06, p=0.042) was significantly positively associated (R^2^=0.278, F_(4,88)_=9.87, p<0.001).

### Behaviour assessment (translation of learning to clinical setting)

The rate of those with opportunities to work with patients with genetic diseases in their practice remained unchanged at 96.4% (or 27/28 people) in the three months after the e-learning course. The implementation rate for behaviour(Appendix 1)is 72.2%-100% (or 13/18-18/18 people) before e-learning and 74.1%-100% (or 20/27-18/18 people) after 3 months of e-learning. The implementation rate increased in the following behaviours: (1) keeping genetic test reports in the Genetic Medicine Department or asking for them to be scanned into electronic medical records with security; (2) having noted in the electronic medical record that you provided genetic counselling (or test explanation) when performing the test; (3) explaining to the patient in advance if there is a possibility that a variant of unknown significance may be found when performing genetic testing; and (4) confirming in advance that the original genetic test result report belongs to the patient when informing them of the genetic test results. However, no statistically significant differences were found for the behaviour questions before and 3 months after the e-learning course.

### Qualitative data

Responses indicate the usefulness of the e-learning course, but also the difficulty of understanding the contents in a single viewing. The importance and challenges of genetic information management were also raised in terms of ethical, legal, and social issues. In the following, we explore the different primary themes and provide clarifying quotes.

### Usefulness of e-learning

‘It was a useful e-learning course’. (No. 10, male, 30s, no clinical experience in genetic medicine, cardiology; No. 35, male, 40s, no clinical experience in genetic medicine, endocrinology and metabolism; No. 74, male, 30s, ≤5 years clinical experience in genetic medicine, oncology; No. 90, male, 50s, ≤5 years clinical experience in genetic medicine, otolaryngology.)

“In a relatively compact time, I was able to learn the ethical knowledge necessary for genetic treatment”. (No. 12, male, 30s, no clinical experience in genetic medicine, cardiology.)

### Difficulty in understanding in one time

“I couldn’t understand all the details just by listening to it once”. (No. 76, female, 30s, ≤5 years clinical experience in genetic medicine, paediatrics.)

### Learning tool issues

“It was difficult to find the video in the e-learning”. (No. 65, female, 50s, no clinical experience in genetic medicine, urology.)

“The test was difficult”. (No. 76, female 30s, ≤5 years clinical experience in genetic medicine, paediatrics.)

### The importance of genetic information management

“It is important to respect the right to privacy and to deal with various ethical issues.” (No. 38, 50s, With clinical experience in genetic medicine within 5 to 10 years, Obstetrics and Gynaecology, Female)

### Issues in the genetic information management

“If there is a template for the procedure of performing genetic testing, it would be easier to omit the description”. (No. 87, female, 40s, 5 to 10 years clinical experience in genetic medicine, breast, thyroid, and endocrine surgery.)

**Table 3:** Physician self-assessment scores regarding their understanding of the medical genetics e-learning course content

Variable	Without clinical experience Mean (SD) n=47	With clinical experience Mean (SD) n=49	Mean Difference (95% CI)	p-value
Genetic medicine department at our hospital	3.26 (0.49)	3.27 (0.45)	0.01 (-0.20, 0.18)	0.963
Basic knowledge of genetic information	3.30 (0.46)	3.24 (0.52)	-0.06 (-0.15,0.25)	0.669
Basic knowledge to perform genetic testing^*1^	3.22 (0.47)	3.25 (0.53)	0.03 (-.24,0.17)	0.704
Genetic testing and genetic counselling in practice^*2^	3.13 (0.58)	3.27 (0.49)	0.14 (-0.36,0.08)	0.232
Genetic information and electronic medical record description	3.17 (0.48)	3.29 (0.46)	0.12 (-0.31,0.08)	0.259
How to access genetic information^*3^	3.13 (0.55)	3.27 (0.49)	0.14 (-0.35,0.08)	0.234
About genetic testing	3.15 (0.51)	3.24 (0.52)	0.09 (-0.31,0.11)	0.364
Considerations and guidelines for genetic medicine	3.21 (0.41)	3.27 (0.45)	0.06 (-0.23,0.12)	0.549

Results of self-assessment scores of understandings of content: Mann-Whitney U test was used to compare the difference between physicians with clinical experience in genetic medicine and those without. *p<0.05 n^*1^=48 (with clinical experience)/46(without clinical experience) n^*2^=48/47 n^*3^=48/45, SD: Standard deviation, CI: Confidence interval   Effect size was estimated using Cohen’s d.

## Discussion

The findings of this study show that our e-learning course on genetic medicine was highly evaluated by about 80% (n = 75-93) of the participants, indicating a high level of satisfaction. Many physicians commented on the usefulness of the e-learning course and the importance genetic information management. Regarding knowledge level, in the objective evaluation, post-course test scores significantly increased, indicating a positive learning effect. In particular, the scores of physicians with no experience in genetic practice increased significantly. However, no significant differences were found in the self-assessment of understanding between those with and without genetic practice experience. No learning effects were evident in the behavioural assessment.

### Effectiveness and Challenges of E-learning seminars

Previous studies have demonstrated that e-learning courses appeared to be an effective strategy for residents’ knowledge acquisition and indicated computer-based learning platforms to be feasible and helpful for the learning processes.^18^ E-learning courses have a number of advantages, such as a uniform format, accessibility, and incentive for regular use.^18^Furthermore, it is generally believed that computer-based instruction offers learner flexibility in terms of controlling and pacing of the learning process, provides for diverse learning needs, gives opportunities for practice through simulation, increases information retention, and reduces instructional time.^19^

In-line with previous reports that attest to the benefits of e-learning, the mean score for the post-test revealed a significant increase from that of the pre-test. The study findings indicate that although there were no differences in self-assessment scores regarding understanding of the course content, post-test scores were significantly higher than pre-test scores, particularly for physicians without experience in genetic practice; years of clinical experience was significantly and positively related to increased scores from pre- to post-test. It was suggested that physicians with no experience in genetic practice may utilize e-learning to acquire new knowledge, while physicians with experience in genetic medicine may find useful ways of learning by considering how education should be based on years of clinical experience. Physicians with fewer years of clinical experience may have limitations in e-learning, suggesting that more practice-based educational methods need to be devised.

American adult educator Malcolm Knowles initially developed the adult learning theory in 1968, with the following key points: Readiness to Learn, Orientation of Learning, Motivation to Learn, Reason to Learn, Self-conceptualization, and Experience.^19^In line with this Knowles’ theory, we found that clinical experience would be associated with learning effectiveness. We found that physicians with no experience in genetic medicine had lower pre-test scores and thus had room to grow, while those already with experience in this field, had high pre-test scores and thus the course offered them little new knowledge. It was suggested that the e-learning course should be divided into different levels with different educational content depending on whether the physician has experience in genetic medicine or not, and provide advanced content for physicians with experience. Previous studies suggest that medical specialists believe that experiential learning in genetic medicine was necessary to develop the confidence and skills needed for clinical care and they expected to look to experts within their own medical specialty who have gained genomics expertise for specific and contextualised support as they develop their knowledge.^20^Learners are said to benefit from communicating with other learners (horizontal communication), from putting questions to experts (vertical communication), and, in the event of technical problems, receiving prompt support.^21^ Clinical teachers have stated that the links between theory and practice would be easier to achieve through dialogue and discussion ^18^of which case studies and group discussion forums are useful for physicians. In summary, it was considered necessary to provide physicians with genetic clinical experience with more educational content relevant to their own clinical speciality, and include experiential and blended learning opportunities. In addition, as genetic medicine involves other professions, the use of interprofessional education may be useful.^22^

### Limitations

This study has several limitations that should be acknowledged. First, owing to the limited number of respondents who responded to the behavioural assessment survey, it is difficult to draw any firm conclusions from our assessment of learning at the behavioural level. Thorough assessment of learning at the behavioural level benefits from long-term observation; therefore, our study design has further limitations in terms of its capacity to adequately assess learning at the behavioural level. Assessment through on-site work, referred to as work-based assessment,^11^ is important, however, reliable assessment methods were considered too difficult to implement in the current study. In addition, the results of our study do not reflect the situation of Japanese healthcare professionals as a whole; the number of non-physician healthcare professionals who took the e-learning course was limited. Therefore, further consideration is needed regarding the provision of a more accessible e-learning system/course.

Future studies should examine how to educate physicians with experience in medical genetics and how to evaluate the learning effects of such education interventions. We should also focus on medical genetics education for non-physician healthcare professionals and consider ways to assess their improvement in genomic literacy.

## Conclusions

In the current study, the usefulness of our e-learning course in medical genetics was demonstrated at a learning level for physicians with no previous experience in medical genetics. For doctors with experience in medical genetics, for whom no significant improvement in post-test scores was observed, it is necessary to provide more practical content and consider different learning methods. This study focussed on assessing the efficacy of our medical genetics e-learning course for physicians; therefore, further studies are required to develop and assess the efficacy of such courses for other medical professionals. Genetic medicine is an issue of growing importance within medical education; however, for busy medical professionals, finding time for continuing education is a challenge. Furthermore, as genomic medicine develops, the learning needs of physicians are expected to change. Therefore, qualitative and quantitative studies are needed to develop and evaluate educational interventions that will meet the evolving needs of physicians with regard to their ongoing education in genetic medicine. The results of this study point to the potential for similar courses to aid medical genetics education for health professionals, which, with developments in our understanding of human genetics and gene therapy, is becoming an ever more important feature of medical care.

### Acknowledgements

The authors wish to thank all medical professionals who participated in this study. We would like to thank the Center for Medical Education and Training of the University of Tsukuba Hospital for creating the E-learning system. We are indebted to Mr T. Mayers, Medical English Communications Center, University of Tsukuba, for grammatical revision of this paper.

### Conflict of Interest

The authors declare that they have no conflicts of interest.

## Supplementary material

Supplementary file 1 Appendix 1. Implementation rates for behaviours before and 3 months after the e-learning course

Supplementary file 2 Appendix 2. Test on Genetic Medicine Pre- and Post-test

Supplementary file 3 Appendix 3. The detailed format of the e-learning course
