# Supplementary material for: Evaluating an e-learning course's impact and challenges on genomic literacy among medical professionals
Source: Int J Med Educ. 2024 Nov 22;15:139–49. doi: 10.5116/ijme.6736.4367 (PMC11774070; doi:10.5116/ijme.6736.4367)
Supplement: Supplementary file 1 — Appendix 1. Implementation rates for behaviours before and 3 months after the e-learning course [file ijme-15-139-S1.pdf]

Appendix 1

Implementation rates for behaviours before and 3 months after the e-learning course

For each of the questions below, please check the box that applies to you at this time.

In the following, genetic tests are defined as germline genetic analysis and chromosome tests not including tests for acquired somatic cell genes such as cancer cells and prenatal diagnosis.

(1) You have the opportunity to work with patients with genetic diseases in your practice.
□ Yes ・ □ No

*If you answered "Yes" to (1), please proceed to (2). If you answered "No," you are done.

(2) When genetic counselling or genetic testing is considered necessary, you consult with a clinical geneticist or refer the patient to a Department of Genetic Medicine.
□ Yes ・ □ No

(3) You are performing genetic testing in your practice.
□ Yes ・ □ No

*If you answered ‘yes’ to (3), please proceed to (4) and the following questions. If you answered ‘No’, you do not need to answer any further questions.

(4) You are providing genetic counselling (or test explanation) using an explanatory document before conducting genetic testing.
□ Yes ・ □ No

(5) When you perform a genetic test, you ask the patient to sign a consent form.
□ Yes ・ □ No

(6) You keep the original genetic test result reports in the Genetic Medicine Department, or you have asked for them to be scanned into your electronic medical record with security.
□ Yes ・ □ No

(7) You have listed the suspected disease name in your electronic medical record when you perform genetic testing.
□ Yes ・ □ No

(8) You are explaining the significance of genetic testing to the recipient when you conduct it.
□ Yes ・ □ No

(9) You have noted in your electronic medical record that you have provided genetic counselling (or test explanation) when you performed the test.
□ Yes ・ □ No

(10) When performing genetic testing, you explain to the patient in advance if there is a possibility that a variant of unknown significance may be found.
□ Yes ・ □ No

(11) When informing patients of the results of genetic testing, you confirm in advance that the original genetic test result report belongs to the patient.
□ Yes ・ □ No

(12) When a genetic diagnosis is confirmed, you record the name of the gene responsible for the disease in the electronic medical record.
□ Yes ・ □ No

(13) For patients diagnosed with genetic diseases, you explain to them and their families the possible effects of the disease on their relatives.
□ Yes ・ □ No
