# Supplementary material for: Evaluating an e-learning course's impact and challenges on genomic literacy among medical professionals
Source: Int J Med Educ. 2024 Nov 22;15:139–49. doi: 10.5116/ijme.6736.4367 (PMC11774070; doi:10.5116/ijme.6736.4367)
Supplement: Supplementary file 2 — Appendix 2. Test on Genetic Medicine Pre- and Post-test [file ijme-15-139-S2.pdf]

Appendix 2

Test on Genetic Medicine Pre- and Post-test

Please check either correct or incorrect answers to the following questions.

Q1
The Personal Information Protection Law revised in 2017 defines 'personal information requiring consideration' as sensitive information, which includes medical history, genetic information and genomic information, and prohibits the acquisition of such information without the prior consent of the individual.

Q2
Genomic data does not fall under the category of 'personal identification code' under the Personal Information Protection Law because it does not contain such an interpretation.

Q3
When the test is performed at an insurance medical institution that has filed a notification pertaining to the facility standard for additional genetic counselling, 1,000 points can be added to the prescribed points as an additional genetic counselling once a month per patient if a doctor with sufficient knowledge of clinical genetics provides the patient with genetic counselling before the test.

Q4
We think that even if genetic test results do not show pathogenic variant in the genes associated with genetic disease A, a clinical diagnosis of genetic disease A does not deny the disease.

Q5
If there is a possibility that a variant of uncertain significance (VUS) will be detected in the results of a genetic test, the patient should not be given an explanation before the genetic test is performed, as this may increase their anxiety.

Q6
The diagnosis can be confirmed if a genetic test is performed on an asymptomatic relative of a patient whose genetic test results show a variant of undetermined significance (VUS) and the same variant is identified.

Q7
The following procedures should be followed when accessing genetic information from electronic medical records and disclosing genetic test results to patients.

a. Identify the patient by asking them to state their name and date of birth.
b. Verify that the name, ID, date of birth, or anonymity code on the result report matches the patient's name on the test request form.
c. Give a copy of the genetic test result report to the patient.

Q8
If a cancer genomic profiling test reveals the possibility of hereditary cancer as secondary findings, the possibility of hereditary cancer should be explained to the patient, whether the patient wants to know the information or not, because it will be useful for the health care of the patient and family.

Q9
If a relative who is clinically suspected of having the same genetic disease as the patient wants a genetic diagnosis, the results of the patient's genetic tests performed in the study may be used to make a genetic diagnosis of the relative, if the patient agrees.

Q10
Companion diagnostics, which may be a diagnosis of a hereditary cancer, do not require genetic counselling prior to genetic testing, as the purpose is to determine whether or not a drug is indicated.

Replacement questions (if you take the test more than once)

Q11
When a relative of a patient with a confirmed genetic diagnosis requests a genetic test, the health care provider may access the patient's genetic test result report and use it for genetic testing of the relative only with the relative's consent.
