# Supplementary figures and images for: Evaluating an e-learning course's impact and challenges on genomic literacy among medical professionals
Source: Int J Med Educ. 2024 Nov 22;15:139–49. doi: 10.5116/ijme.6736.4367 (PMC11774070; doi:10.5116/ijme.6736.4367)

Appendix 3

The detailed format of the e-learning course

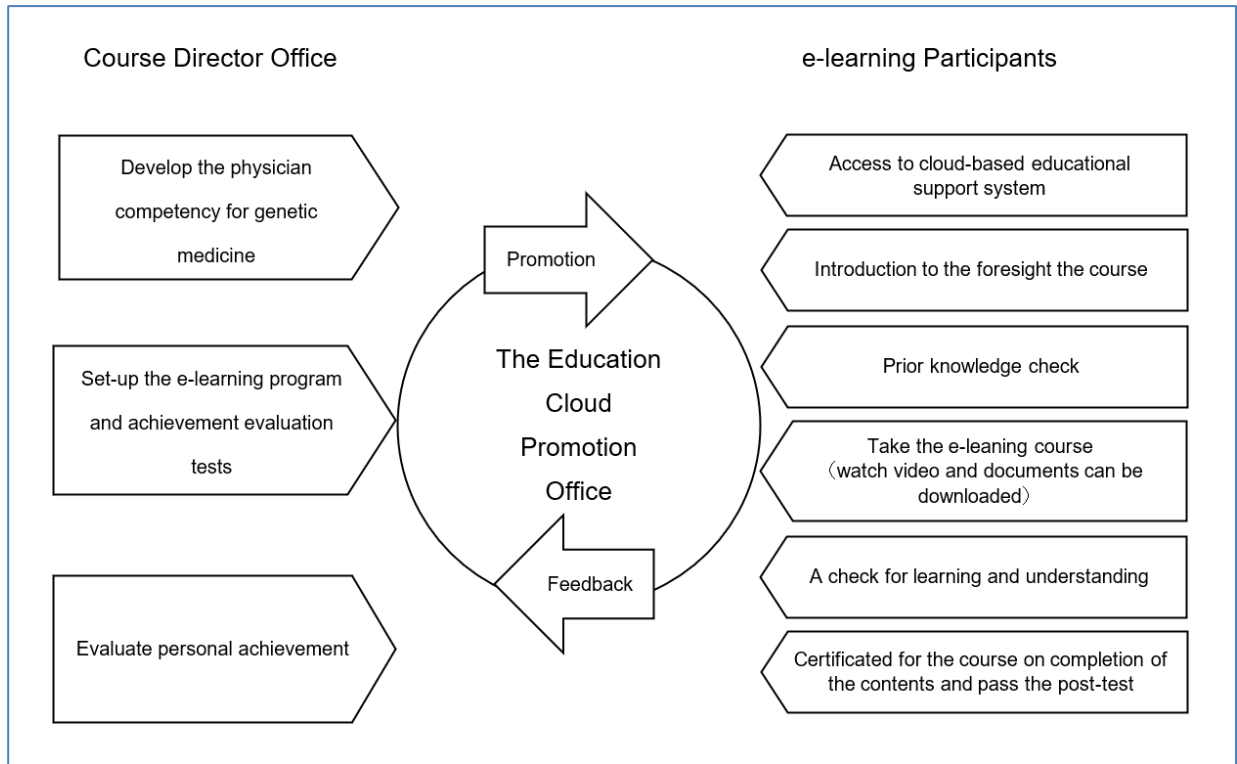

Supplement: Supplementary file 3 — Appendix 3. The detailed format of the e-learning course [file ijme-15-139-S3.pdf]
